# Zebrafish Bioassay-Guided Natural Product Discovery: Isolation of Angiogenesis Inhibitors from East African Medicinal Plants

**DOI:** 10.1371/journal.pone.0014694

**Published:** 2011-02-17

**Authors:** Alexander D. Crawford, Sandra Liekens, Appolinary R. Kamuhabwa, Jan Maes, Sebastian Munck, Roger Busson, Jef Rozenski, Camila V. Esguerra, Peter A. M. de Witte

**Affiliations:** 1 Department of Pharmaceutical Sciences, University of Leuven, Leuven, Belgium; 2 Rega Institute for Medical Research, University of Leuven, Leuven, Belgium; 3 Department of Microbiology and Immunology, University of Leuven, Leuven, Belgium; 4 Department of Pharmacognosy, Muhimbili University of Health and Allied Sciences, Dar es Salaam, Tanzania; 5 Department of Human Genetics, Flanders Interuniversity Institute of Biotechnology, University of Leuven, Leuven, Belgium; Katholieke Universiteit Leuven, Belgium

## Abstract

Natural products represent a significant reservoir of unexplored chemical diversity for early-stage drug discovery. The identification of lead compounds of natural origin would benefit from therapeutically relevant bioassays capable of facilitating the isolation of bioactive molecules from multi-constituent extracts. Towards this end, we developed an *in vivo* bioassay-guided isolation approach for natural product discovery that combines bioactivity screening in zebrafish embryos with rapid fractionation by analytical thin-layer chromatography (TLC) and initial structural elucidation by high-resolution electrospray mass spectrometry (HRESIMS). Bioactivity screening of East African medicinal plant extracts using *fli-1*:EGFP transgenic zebrafish embryos identified *Oxygonum sinuatum* and *Plectranthus barbatus* as inhibiting vascular development. Zebrafish bioassay-guided fractionation identified the active components of these plants as emodin, an inhibitor of the protein kinase CK2, and coleon A lactone, a rare abietane diterpenoid with no previously described bioactivity. Both emodin and coleon A lactone inhibited mammalian endothelial cell proliferation, migration, and tube formation *in vitro*, as well as angiogenesis in the chick chorioallantoic membrane (CAM) assay. These results suggest that the combination of zebrafish bioassays with analytical chromatography methods is an effective strategy for the rapid identification of bioactive natural products.

## Introduction

Small molecules from natural sources are recognized as evolved, privileged structures with greater likelihood than many synthetic compounds to exhibit specific bioactivities [1]. For example, 73% of cancer therapeutics approved to date are either natural products or derivatives thereof [2]. Nevertheless, the use of natural products in drug discovery has significantly declined in the past two decades, due in part to persisting difficulties in the systematic isolation and synthesis of such molecules [3]. One promising strategy to better exploit the therapeutic potential of natural products could be the use of more biomedically relevant assays – ideally *in vivo* models – for the screening and bioactivity-guided fractionation of plant, fungal and microbial extracts.

Many currently known bioactive natural products were originally identified using *in vitro* assays (e.g. cytotoxicity in tumor cells) for their activity-guided isolation from extracts. The biological activity of many other natural products was determined only after their initial isolation on the basis of physical characteristics (e.g., preparative chromatography followed by mass spectrometry and NMR spectroscopy analysis). Because of the low throughput of conventional *in vivo* models such as mice and rats, in addition to the relatively large amounts of compound required for testing in these systems, *in vivo* assay-guided fractionation is currently not a widely-used approach for the discovery of drug-like natural products.

Novel opportunities for *in vivo* natural product discovery have arisen through the recent emergence of zebrafish as an effective model system for the identification of disease-relevant genes and bioactive small molecules [4]. Large-scale genetic screens in zebrafish carried out since the early 1990s have led to the identification of therapeutically relevant genes in several indication areas, including cardiovascular, neurological, gastrointestinal, musculoskeletal, and metabolic disorders [5], [6], [7], [8]. In addition, small-molecule screens carried out in zebrafish within the past decade have confirmed the ability of this model system to identify bioactive compounds in a target-independent manner, thereby enabling the discovery of novel mechanisms of action [9], [10], [11], [12], [13].

The primary advantages of zebrafish for drug discovery include their high genetic, physiologic, and pharmacologic similarity with humans, as well as the small size, optical transparency, rapid development, and large numbers of their embryos and larvae, which are the primary system for experimental analysis. Because of their small size (1 to 5 mm), zebrafish embryos and larvae are compatible with microtiter plates for screening (primarily 24- and 96-well plates, but even 384-well plates are possible), thereby requiring only microgram amounts of each extract, fraction, or compound to be tested. Because of the high fecundity of zebrafish, large numbers of embryos and larvae can be produced and analyzed in a more cost-effective manner than, for example, mice and rats. Combined, these features define zebrafish as an ideal *in vivo* model for the systematic identification of bioactive natural products with therapeutic potential [4].

For an initial evaluation of zebrafish as a platform for natural product discovery, we opted to identify novel inhibitors of angiogenesis. Despite the recent regulatory approval of recombinant antibodies and small molecules targeting the vascular endothelial growth factor (VEGF) pathway, the clinical efficacy of these therapies for various cancers is limited [14]. Also, despite the large number of compounds targeting this pathway [15], many of these have shown limited or insufficient efficacy in clinical trials, or are associated with toxicities such as arterial thromboembolic events [16]. For these reasons, there is still a need for novel anti-angiogenic compounds with different mechanisms of action, some of which might be suitable for use in combinatorial therapy strategies [17].

Numerous *in vivo* and *in vitro* assays have been developed since the 1970s for the evaluation of anti-angiogenic molecules [18], yet because of various disadvantages (low throughput, high cost, and the requirement for larger compound amounts in the case of *in vivo* assays, and limited predictive value in the case of *in vitro* assays), these are not ideal as front-line assays for natural product discovery (i.e. for high-throughput screening and rapid bioassay-guided fractionation). Zebrafish, however, offer an interesting combination of (1) being an *in vivo* model and (2) enabling high-throughput, low-volume screening. Within the past decade, zebrafish embryos have become well-established as an *in vivo* model for the analysis of angiogenesis and vascular development [9], [19], [20], [21], [22], [23]. To test the suitability of zebrafish as an *in vivo* frontline assay for the bioassay-guided fractionation of complex natural extracts, we therefore combined an embryonic zebrafish angiogenesis assay with analytical chromatography methods, with the goal of rapidly isolating phytochemicals from medicinal plant extracts capable of inhibiting vascular outgrowth in this assay.

## Results and Discussion

**Figure 1:**
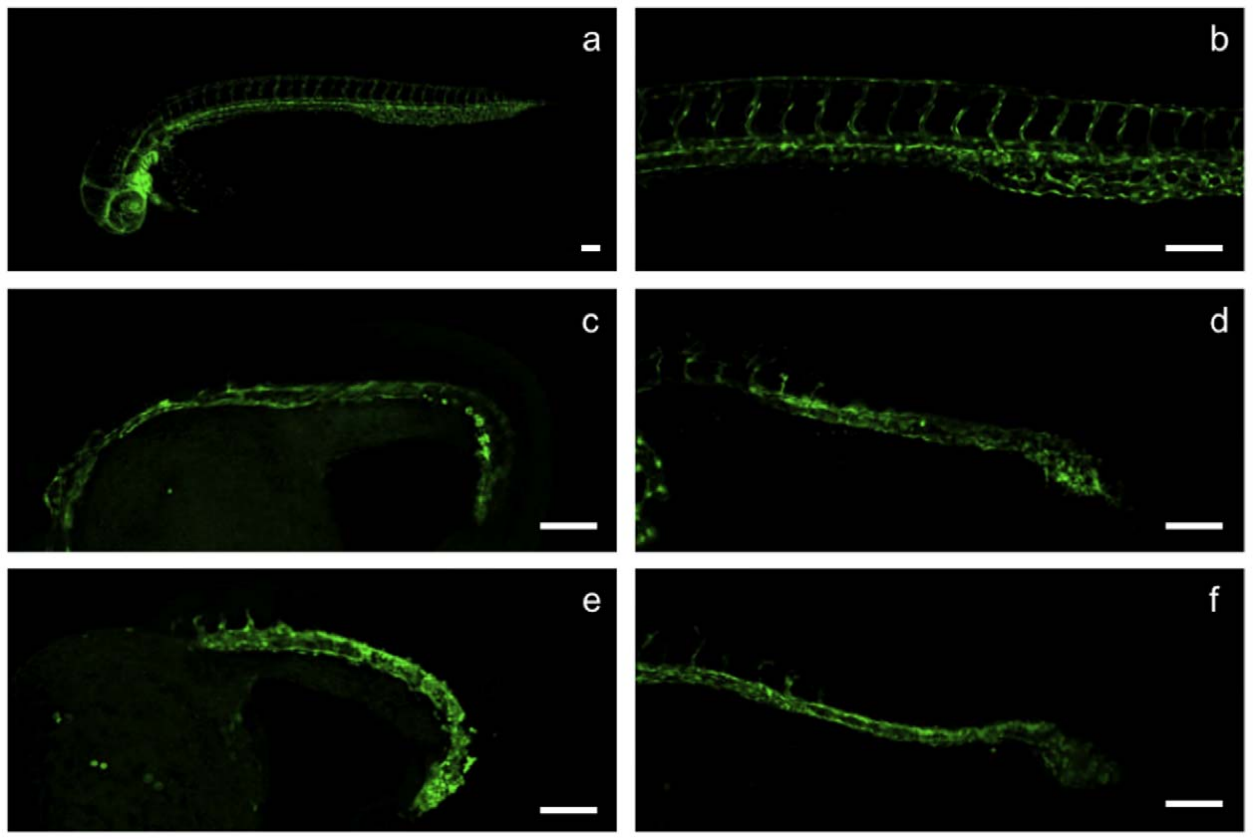
Crude methanolic extracts and isolated compounds inhibit vascular outgrowth in *fli-1*:EGFP transgenic embryos. All embryos are 40 hpf, with anterior to the left, scale bar  =  100 µm. a, b, DMSO-treated control; c, embryo treated with 200 µg/ml *O. sinuatum* extract; d, embryo treated with 10 µg/ml *P. barbatus* extract; e, embryo treated with 8 µM emodin; f, embryo treated with 2 µM coleon AL.

We chose an angiogenesis assay based on the evaluation of intersegmental vessel (ISV) outgrowth in *fli-1*:EGFP transgenic embryos [19], which exhibit vasculature-specific expression of enhanced green fluorescent protein (EGFP) in the trunk and tail during embryonic and larval development (Figs. 1a, b). With respect to natural product research, *fli-1*:EGFP zebrafish have been used to characterize the angiogenic activity of *Angelica sinensis* (dong quai) [24], as well as the anti-angiogenic activity of solenopsin, an alkaloid isolated from *Solenopsis invicta* (fire ants) [25]. Similar transgenic lines, with fluorescent reporter proteins expressed under the control of the endothelial cell-specific *flk-1*/*VEGFR2* promoter, have recently enabled an ENU mutagenesis screen to identify genetic determinants of vascular development [26] and a small-molecule screen to identify novel angiogenesis inhibitors [27].

**Figure 2:**
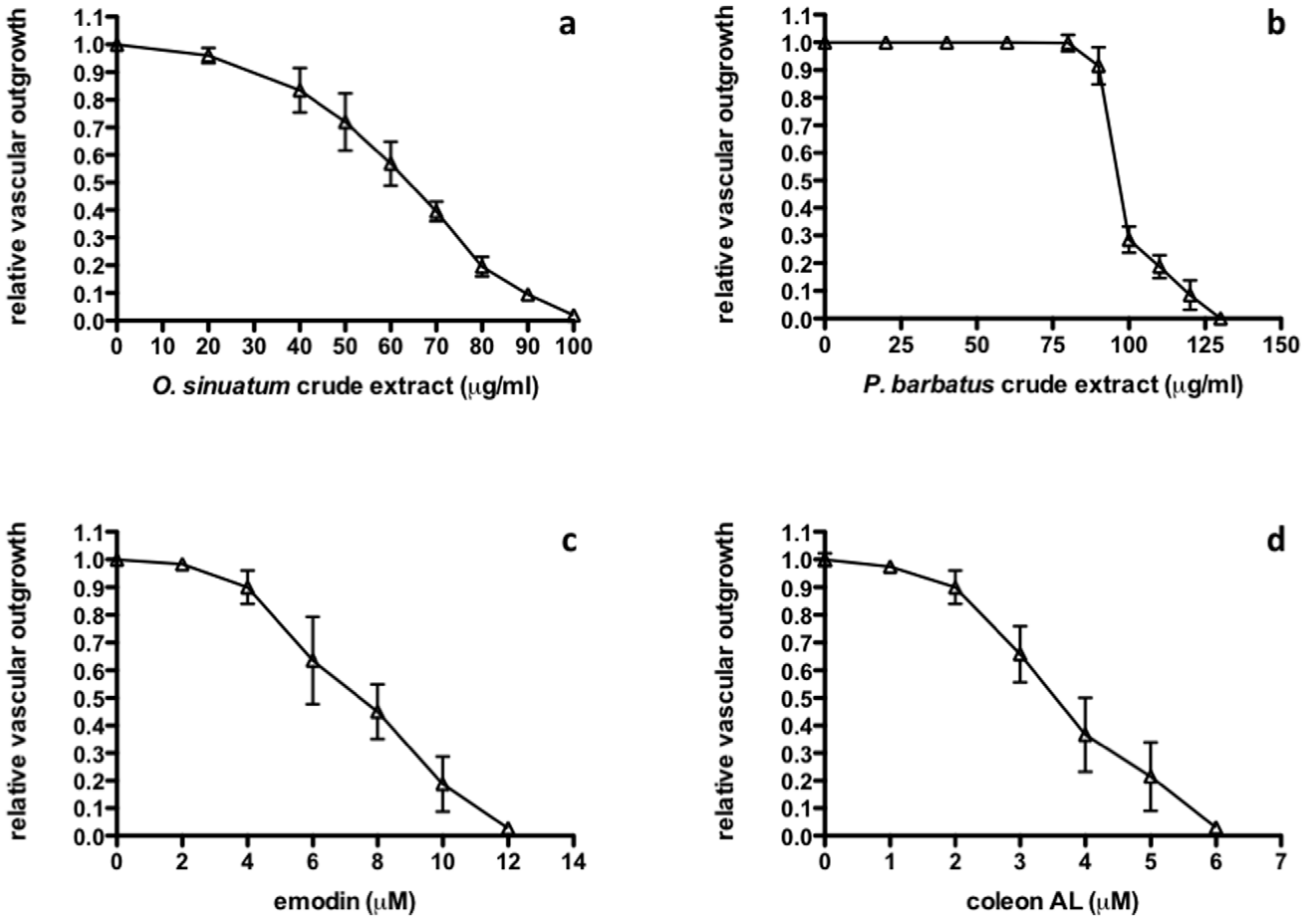
Crude methanolic extracts and isolated compounds exhibit concentration-dependent effects on vascular outgrowth in zebrafish embryos. Relative vascular outgrowth scores are shown (see Materials & Methods). a, *O. sinuatum* crude extract; b, *P. barbatus* crude extract; c, emodin; d, coleon AL.

To test the utility of this zebrafish assay for natural product discovery, we screened crude methanolic extracts from over 80 East African medicinal plants. Two extracts, from *Oxygonum sinuatum* (Meisn.) Dammer (Polygonaceae) and *Plectranthus barbatus* Andrews (Lamiaceae), inhibited ISV outgrowth in *fli-1*:EGFP embryos in a dose-dependent manner (Figs. 1c, d and 2a, b).

In terms of known bioactivities for these plants, *O. sinuatum* has been documented as an ethnobotanical treatment in Kenya for several unrelated disorders [28]. No phytochemical analysis of this plant has been reported to date. *P. barbatus* (previously also known as *Coleus forskohlii* Briq.) is widely used in traditional medicine in Africa and Latin American to treat a range of human ailments [29]. This species is also well-known as the primary source of forskolin, a labdane diterpenoid and activator of cAMP signaling [30], [31]. Intruigingly, although forskolin has been shown to inhibit angiogenesis in the chick chorioallantoic membrane assay [32] and *in vitro* [33], it is also known to upregulate VEGF expression [34], making its overall effect on angiogenesis *in vivo* difficult to predict. We determined that forskolin does not inhibit angiogenesis in zebrafish (data not shown) and since it is isolated from *P. barbatus* roots (an extract from leaves being the subject of this analysis), we concluded that the anti-angiogenic activity seen in zebrafish embryos for the *P. barbatus* extract is likely due to the bioactivity of another compound.

**Figure 3:**
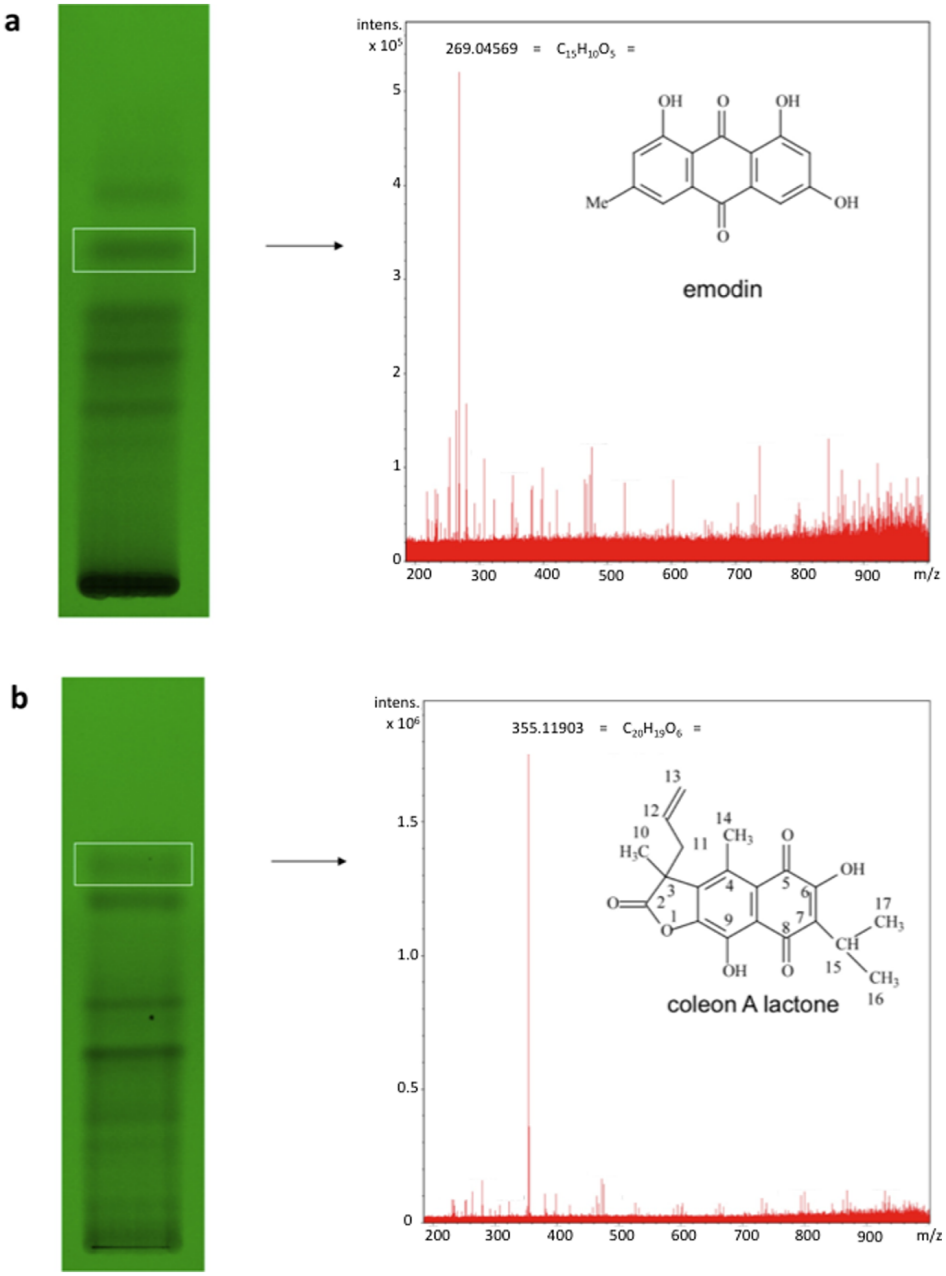
TLC fractionation and HRESIMS analysis identify emodin and coleon AL as bioactive constituents. Thin-layer chromatograms, HRESIMS spectra, and predicted structures of isolated, bioactive compounds. a, *O. sinuatum*; b, *P. barbatus*.

We next sought to isolate from *O. sinuatum* and *P. barbatus* extracts the principle components responsible for their anti-angiogenic effects. Both crude methanolic extracts were fractionated via thin-layer chromatography (TLC), using toluene/ethyl formate/formic acid (5:4:1) as the solvent. A single analytical-scale TLC plate (20×20 cm) was used to separate 10 mg of each extract, and was subsequently divided into 10-15 horizontal strips based on the presence of UV_254_ -absorbing and UV_365_-emitting components (Figs. 3 a, b). The silica was removed from these strips and extracted with methanol, after which the eluted constituents were subjected to bioactivity analysis in zebrafish, followed by high-resolution electrospray ionization mass spectroscopy (HRESIMS) for those exhibiting anti-angiogenic activity. For both *O. sinuatum* and *P. barbatus*, single TLC fractions were identified in this manner which phenocopied the anti-angiogenic activity shown by the crude extracts.

O. sinuatum yielded a single bioactive compound whose molecular formula was determined to be C_15_H_10_O_5_ based on the deprotonated molecular ion at m/z 269.0457 identified by HRESIMS analysis (negative mode), suggesting 6-methyl-1,3,8-trihydroxyanthraquinone (emodin) – a known product of several other Polygonaceae species. Emodin has recently been reported as an inhibitor of angiogenesis in vitro [35] and in vivo [36], and has been shown to be an inhibitor of the protein kinases Lck [37], HER-2 [38], and CK2 [39]. More recently, emodin was shown to inhibit angiogenesis in vitro at least in part by restricting the phosphorylation of VEGFR2 [40]. In addition; CK2 has been found to directly phosphorylate Akt [41], one of several downstream elements of VEGF signaling, and this modification has been shown to be essential for nuclear retention of FOXO1, an important cytoplasmic inhibitor of angiogenesis [42]. Semi-synthetic emodin revealed an MS spectrum identical to the bioactive compound isolated from O. sinuatum (data not shown) and, importantly, phenocopied both this compound and the crude extract (Fig. 1e, 2c), thereby confirming emodin as the primary constituent responsible for this plant's bioactivity. Furthermore, emodin and other anthraquinones synthesized by Rheum species (rhubarb root, or Dahuang) have recently also been shown to inhibit vascular outgrowth in zebrafish using a histochemical assay to visualize blood vessels [43].

*P. barbatus* yielded a bioactive molecule with an apparent *M_w_* of 355.1190 based on HRESIMS analysis and the predicted molecular formula C_20_H_19_O_6_, suggesting coleon A lactone, a known product [44] of another Lamiaceae species but with no previously described bioactivity. Following isolation by preparative-scale chromatography, the identity of coleon A lactone (hereinafter referred to as coleon AL) was confirmed by NMR (Table 1).

**Table 1:** NMR spectroscopy analysis confirms identity of coleon AL.

a. ^1^H NMR data in CDCl_3_		
Ref. 44		experimental
1.31 (6H, d, J = 7.1 Hz)	CH_3_-16/17	1.306 (6H, d, J = 7.0 Hz)
1.65 (3H, s)	CH_3_-10	1.662 (3H, s)
2.69 (3H, s)	CH_3_-14	1.689 (3H, s)
2.83 (2H, d, J = 6.0 Hz)	H-11AH-11B	2.811 (1H, dd, ^2^J = 14.0 Hz, ^3^J = 7.5 Hz)2.868 (1H, dd, ^2^J = 14.0 Hz, ^3^J = 7.3 Hz)
3.38 (1H, pent, J = 7.1 Hz)	H-15	3.378 (1H, sept, J = 7.0 Hz)
4.90 – 5.60 (3H, m)	H-13AH-13BH-12	4.955 (1H, dd, J = 9.9 Hz, J = 1.1 Hz)5.054 (1H, dd, J = 17.0 Hz, J = 1.1 Hz)5.326 (1H, ddt, J = 17.0/9.9/7.4 Hz)
7.96 (1H, s)	9-OH	8.01 (1H, s)
13.25 (1H, s)	6-OH	13.35 (1H, s)

b. ^13^C NMR data in CDCl_3_
190.8 (C-8), 181.3 (C-5), 177.7 (C-2), 154.1 (C-6), 146.9 (C-9a), 146.1 (C-9), 135.5 (C-3a), 132.0 (C-4a), 130.8 (C-12), 126.4 (C-7), 122.5 (C-4), 120.2 (C-13), 117.1 (C-8a), 50.0 (C-3), 41.6 (C-11), 23.9 (C-15), 22.9 (C-10), 19.7 (C-16/17), 17.3 (C-14) ppm.

a, Comparison of 1H-NMR chemical shift assignments from Ref. 44 and our experimental data, with more resolution for protons H-11A, 11B, 13A, 13B and 12; b, 13C NMR experimental chemical shift assignments, with 3 carbonyl peaks at 177–190 ppm, and olefinic peaks at 117–154 ppm corresponding to the aromatic fused-ring moeities.

**Figure 4:**
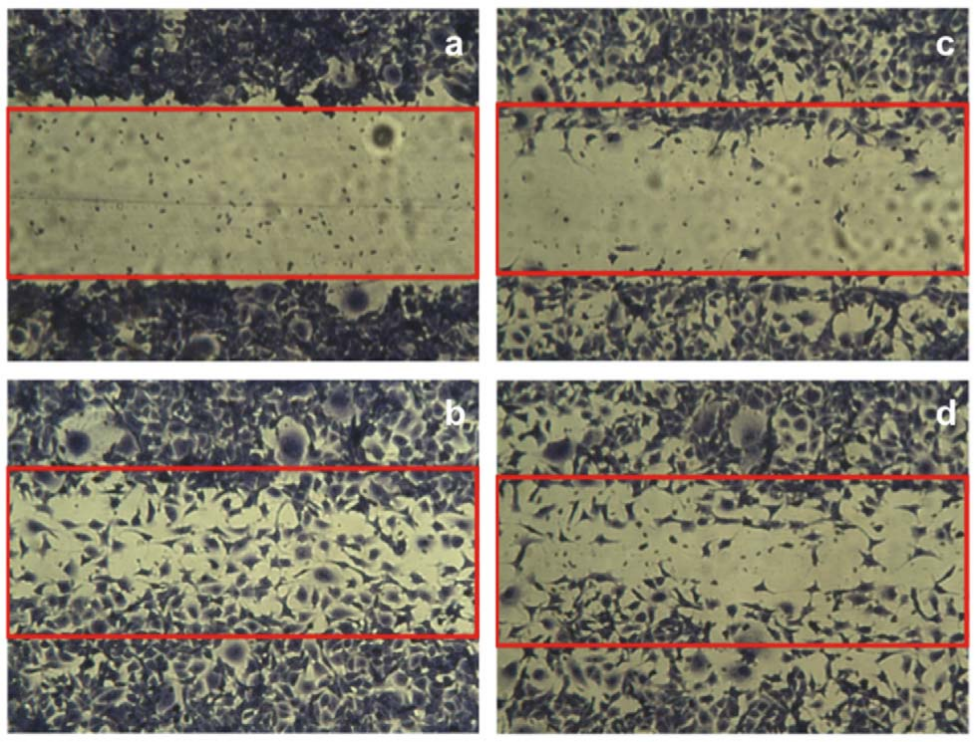
Emodin and coleon AL inhibit endothelial cell migration *in vitro*. a, control (immediate); b, control; c, 30 µM emodin; d, 30 µM coleon AL.

**Figure 5:**
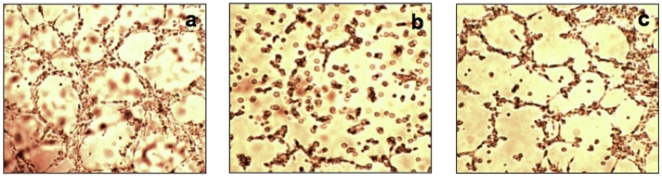
Emodin and coleon AL inhibit endothelial tube formation *in vitro*. a, DMSO-treated control; b, 10 µM emodin; c, 10 µM coleon AL.

**Figure 6:**
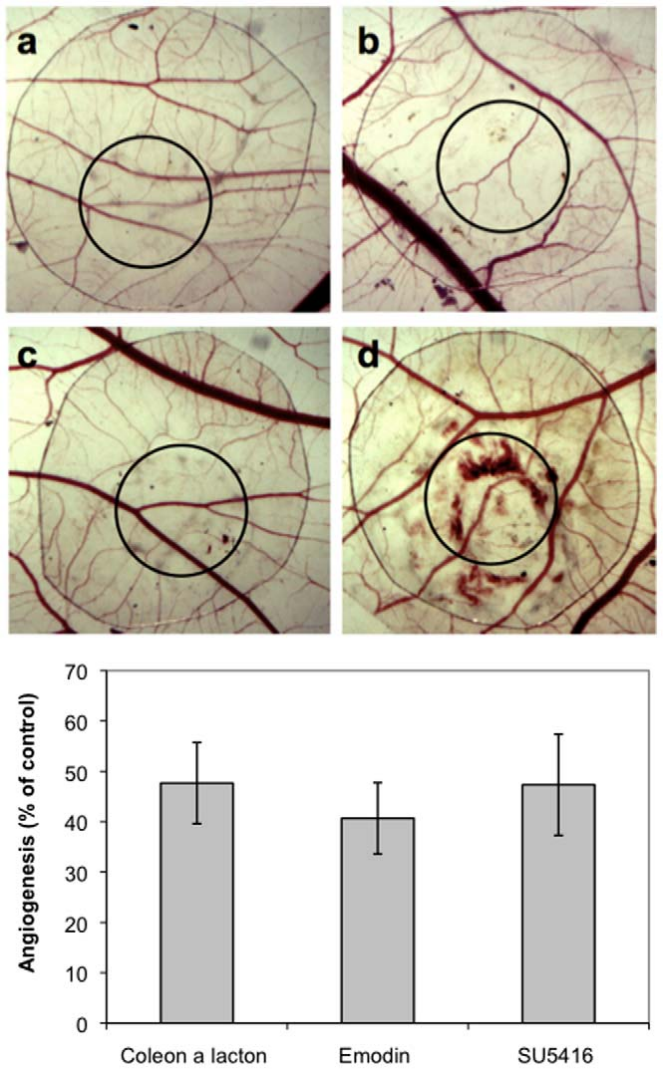
Emodin and coleon AL inhibit angiogenesis in the chick chorioallantoic membrane (CAM) assay. a, negative control; b, positive control (50 nmol SU5416); c, 50 nmol emodin; d, 50 nmol coleon AL.

Zebrafish have only recently been utilized for the systematic identification of bioactive small molecules [12], so the predictive power of zebrafish assays for drug discovery will only become clear as molecules found using this platform are advanced into the clinic. In any case, to further evaluate the therapeutic potential of natural products identified using embryonic or larval zebrafish models, bioactive compounds should subsequently be validated using a series of additional *in vitro* and *in vivo* assays. To this end, *in vitro* anti-angiogenesis assays were carried out to further characterize the anti-angiogenic activity of the bioactive natural products isolated in this study, revealing both emodin and coleon AL to inhibit the proliferation, migration and tube formation of mammalian endothelial cells (Table 2, Fig. 4, 5). In addition, both compounds inhibited blood vessel formation in the chick chorioallantoic membrane (CAM) assay (Fig. 6). Emodin and coleon AL inhibited the proliferation of mouse aortic endothelial cells (MAECs) with an IC_50_ similar to that of the vascular endothelial growth factor (VEGF) receptor inhibitor SU5416, a synthetic indoline derivative [45], and inhibited the proliferation of bovine aortic endothelial cells (BAECs) with an IC_50_ similar to that of the PI3K inhibitors wortmannin, a fungal furanosteroid [46], and LY294002, a synthetic chromone derivative [47]. In the CAM assay, we determined the anti-angiogenic activity of emodin and coleon AL to be similar to that of SU5416.

**Table 2:** Emodin and coleon AL inhibit endothelial cell proliferation *in vitro*.

	IC_50_ (µM)	
	MAEC	BAEC
emodin	32±11	39±9
coleon AL	30±5	31±13
wortmannin	55±5	29±12
LY294002	7±2	3±1
SU5416	29±2	8±2

IC_50_ values (± SD) for emodin and coleon A lactone are shown for the inhibition of proliferation of both bovine aortic endothelial cells (BAEC) and mouse aortic endothelial cells (MAEC). For comparison, effects of 3 other anti-angiogenic compounds are shown (wortmannin, LY294002, and SU5416).

As a next step, *in vivo* angiogenesis assays should be carried out for these compounds in mammalian models, including mouse tumor assays [18]. Additional *in vitro* assays should also be performed to determine the activity of anti-angiogenic compounds under hypoxic conditions, an important condition for possible application in antitumor therapy [48].

Zebrafish were first proposed as a model for *in vivo* drug discovery in 1957 by Jones and Huffmann of the Oklahoma Research Foundation [49], and soon thereafter were used for the first time to analyze the bioactivities of natural products [50]. Only more recently, however, have zebrafish been widely used for the function-based identification of disease-relevant genes and bioactive compounds [12]. An important advantage of using zebrafish embryos and larvae for the identification of bioactive molecules is that they are living organisms, thereby enabling the rapid *in vivo* evaluation of compounds not only in terms of their pharmacological activity but also of their possible toxicity (particularly cardio-, neuro-, and hepatotoxicity) early in the drug discovery process [51], [52]. Within the past decade, zebrafish have furthermore emerged as a powerful model for chemical genetics, both with respect to the *in vivo* dissection of signaling pathways [20], [53] and to the elucidation of the mechanism of action of bioactive natural products [54], [55]. In the area of natural product discovery, one recent report describes the application of a histochemical assay in zebrafish to guide the isolation of anti-angiogenic terpenoids from *Tripterygium wilfordii*, an anti-inflammatory Chinese medicinal plant, using preparative chromatography methods [56].

Here, we demonstrate the utility of zebrafish bioassay-guided fractionation by analytical chromatography techniques, and further establish zebrafish as an *in vivo* platform for the discovery of bioactive natural products. Based on these initial results, it appears possible that zebrafish can help address a critical bottleneck in natural product discovery by enabling the rapid, *in vivo* and microgram-scale bioactivity analysis, bioassay-guided fractionation, and dereplication of complex natural extracts. While data described here were obtained using zebrafish bioassay-guided TLC fractionation, additional advantages for accelerating natural product discovery will be realized through the combination of zebrafish bioassays with more advanced HPLC techniques – in particular, those enabling microfractionation [57], [58]. With a wide array of biomedically relevant assays now established in zebrafish [4], the advantages of this *in vivo* system for natural product discovery should facilitate the systematic identification of a new generation of bioactive natural products with potential utility in drug discovery.

## Materials and Methods

### Zebrafish

The *fli-1*:EGFP transgenic line [19] was obtained from the Zebrafish International Resource Center at the University of Oregon (Eugene, Oregon, USA). Zebrafish husbandry, embryo collection, and embryo and larvae maintenance were performed as described earlier [59], [60]. Zebrafish assays were standardly performed in 24-well microtiter plates using 10 embryos per well in 1 ml of 0.3× Danieau's medium (17 mM NaCl, 2 mM KCl, 0.12 mM MgSO_4_, 1.8 mM Ca(NO_3_)_2_ and 1.5 mM HEPES, pH 7.6). Embryos were exposed to extracts and compounds at 16 hours post-fertilization (hpf) – approximately 8 hours prior to the initiation of intersegmental vessel (ISV) outgrowth – and scored for relative vascular outgrowth at 40 hpf. Extracts and compounds were solubilized in dimethyl sulfoxide (DMSO, Agros Organics, Geel, Belgium), and were added to the medium up to a maximum DMSO concentration of 1%. The extent of outgrowth of intersegmental vessels (ISVs) was determined using a scoring method that takes into account both the approximate number of outgrowing vessels (100, 75, 50, 25, or 0%) and the average degree to which these vessels have extended into the trunk from the dorsal aorta/posterior cardinal vein (DA/PCV) (100, 75, 50, 25, or 0%). These two values are multiplied to give the relative vascular outgrowth (RVO) score.

### Extracts

Plant samples were collected from different locations in Tanzania and their respective voucher specimens deposited at the Department of Pharmacognosy, Faculty of Pharmacy of the Muhimbili University of Health and Allied Sciences (MUHAS), Dar es Salaam, Tanzania. For each plant sample, plant materials were dried at room temperature and ground. The dry, powdery plant samples were exhaustively extracted with methanol by maceration. Dry methanolic extracts were obtained after removing the solvent by evaporation under reduced pressure. Prior to testing, aliquots of each dry methanolic extract were suspended in 100% DMSO; these stock solutions were then kept at −20 °C.

### Compounds

Semi-synthetic emodin was obtained from Janssen Chimica (Geel, Belgium). SU5416 and wortmannin were obtained from Sigma-Alrich (Bornem, Belgium) and LY294002 was obtained from Cayman Chemical (Talinn, Estonia). Coleon A lactone (coleon AL) was isolated from leaves of *Plectranthus barbatus* collected in Handeni, Tanga Region, Tanzania. Leaves were dried at ambient temperature under sunlight, homogenized, and extracted 3 times with chloroform (1 l chloroform per 100 g leaves). This chloroform extract was concentrated 100:1 on a rotary evaporator and subjected to chromatographic separation on a LaFlash chromatography apparatus from VWR (West Chester, Pennsylvania, USA) using VWR SuperVarioFlash silica cartridges (30 g, Si60, pore size 15-40 µM), with chloroform:acetic acid 200:1 as the solvent. Coleon AL was isolated as the second major peak showing absorbance at 254 nm.

### Thin-layer chromatography

TLC plates were obtained from Macherey-Nagel (Düren, Germany). For the experiments described here, 20×20 cm aluminum plates coated with TLC silica gel 60 (layer thickness, 0.2 mm) containing a UV_254_ fluorescence indicator were used (ALUGRAM SIL G/UV_254_, Machery-Nagel product number 818133). Plates were loaded manually, using a finely tapered micropipette tip, with 10 mg of crude extract (50 mg/ml in methanol), dried for 15 seconds with a hair dryer at low heat, and placed in an enclosed, upright 25×25×10 cm glass chamber containing 100 ml toluene/ethyl formate/formic acid 5:4:1 (pre-equilibrated for at least 15 minutes, and with 3 vertical faces covered with solvent-saturated filter paper).

### High-resolution electrospray ionization mass spectrometry

Electrospray ionization (ESI) mass spectra were recorded in positive and negative mode on an orthogonal acceleration quadrupole time-of-flight mass spectrometer (Q-Tof 2, Micromass, Manchester, UK). The electrospray needle voltage was set to 3000 V or −2850 V for the positive and negative mode respectively. Fragment ion spectra were obtained by selecting the precursor ion in the quadrupole and collisional activation with argon gas in the collision cell. Accurate mass measurements were performed at a resolution of 9000 using the protonated leucine-enkephaline (YGGFL) ion as lock mass.

### NMR spectroscopy

^1^H and ^13^C NMR spectra were recorded on a Bruker (Fällanden, Switzerland) Avance II 500 spectrometer operating at 500.130 MHz for ^1^H and at 125.758 MHz for ^13^C, and using a gradient-equipped inverse 5 mm triple probe with π/2 pulses of 6.5, and 14.5 µs respectively. The standard Bruker Topspin 2.1 software under Windows XP was used throughout. All experiments were performed at 22 °C in deuterochloroform solution with the solvent peak as internal standard set at 7.27 ppm (^1^H) or 77.0 (^13^C) vs. TMS respectively. First-order analysis was applied throughout, and first-order multiplets or apparent first-order multiplets were denoted as follows: s  =  singlet, d  =  doublet, dd  =  double doublet, t  =  triplet. J-values were extracted directly from the splittings in the spectrum, and are not optimised. Spectral assignments were based not only on the usual chemical shift rules and coupling patterns, but especially on routine 2D-correlations such as COSY45- (homonuclear H,H J-correlations), GHSQC- (single bond C,H ^1^J-correlations) and GHMBC-experiments (multiple bond C,H ^3^J-correlations). The data for coleon AL are summarized in Fig. 4 and compared with previously reported values [44].

### Imaging

Zebrafish were screened for GFP fluorescence using an Axiovert 40 CFL microscope from Zeiss (Oberkochen, Germany) equipped with an MBQ 52 AC fluorescence lamp from LEJ (Jena, Germany). Micrographs of zebrafish embryos were taken on a Stemi 2000 stereo microscope from Zeiss equipped with a DP200 CMOS digital camera and using DpxView Pro EE EF software, both from Deltapix (Maalov, Denmark). Confocal fluorescence micrographs of zebrafish embryos were acquired using a Nikon A1R confocal unit mounted on a Ti2000 inverted microscope (Nikon, Tokyo, Japan). The microscope was equipped with 4× (0.2 N.A.) and 10× (0.45 N.A.) objective lenses, and fluorescence was revealed using a 488 nm laser line (CVI Melles Griot, Albuquerque, NM, USA). For imaging, zebrafish embryos were anesthetized using 0.1 mg/ml ethyl 3-aminobenzoate methanesulfonate (Sigma) in 0.3× Danieau's solution.

### Cell cultures

Mouse aortic endothelial cells (MAEC) and bovine aortic endothelial cells (BAEC) were kindly provided by Prof. M. Presta (Brescia, Italy). The cells were grown in Dulbecco's modified minimum essential medium (DMEM, Life Technologies, Rockville, MD, USA) supplemented with 10 mM Hepes (Life Technologies) and 10% fetal calf serum (FCS, Harlan Sera-Lab, Loughborough, UK).

### Cell proliferation assays

Cells (MAEC or BAEC) were seeded in 48-well plates at 10,000 cells per cm^2^. After 16 h, the cells were incubated in fresh medium in the presence of different concentrations of the test compounds (i.e. 100 - 20 - 4 - 1 - 0.2 µM). On day 5, cells were trypsinized and counted by a Coulter counter (Analis, Belgium). The compound concentration that inhibits cell growth by 50 % (i.e. IC_50_) was calculated based on cell counts in control cultures.

### Cell migration assay

Wounds were created in confluent MAE cell monolayers with a 1.0-mm wide micropipette tip. Then, cells were incubated in fresh medium containing 10% FCS in the presence of the test compounds. After 8 h, the wounds were photographed, and endothelial cells invading the wound were quantified by computerized analysis of the digitalized images.

### Tube formation assay

Wells of a 96-well plate were coated with 60 µl matrigel (10 mg/ml, BD Biosciences, Heidelberg, Germany) at 4 °C. After gelatinization at 37 °C during 30 min, BAEC (60,000 cells) were seeded on top of the matrigel in 200 µl DMEM containing 1% FCS and the test compounds. After 6 hours of incubation, the cell structures were photographed at 100×magnification. Tube formation was quantified by counting the number of branching points.

### Chorioallantoic membrane (CAM) assay

The *in vivo* CAM angiogenesis model was performed as described with slight modifications [61]. Fertilized chicken eggs were incubated for 3 days at 37 °C when 3 ml of albumen was removed (to detach the shell from the developing CAM) and a window was opened on the eggshell exposing the CAM. The window was covered with cellophane tape and the eggs were returned to the incubator until day 9 when the compounds were applied. The compounds were placed on sterile plastic discs (Ø 8 mm), which were allowed to dry under sterile conditions. A solution of cortisone acetate (100 µg/disc, Sigma-Aldrich, St. Louis, MO, USA) was added to all discs in order to prevent an inflammatory response. A loaded and dried control disc was placed on the CAM approximately 1 cm away from the disc containing the test compound(s). Next, the windows were covered and the eggs further incubated until day 11 when the area around the discs was cut-off and photographed. Then, 2 concentric circles were positioned on the digitalized pictures and all vessels intersecting these circles were counted. A two-tailed paired Student's *t*-test was performed to assess the significance of the obtained results.
